# Fall-risk-increasing drug use in older community-dwelling adults: prevalence, association with fall-related admissions and post-discharge prescribing changes—a retrospective cohort study

**DOI:** 10.1093/ageing/afag303

**Published:** 2026-10-23

**Authors:** Steven Gilmore, Ann Sinéad Doherty, Frank Moriarty, Fiona Boland, Caroline McCarthy, Ryan Muddiman, Barbara Clyne, Tom Fahey, Denis O’Mahony, Emma Wallace

**Affiliations:** Department of General Practice, School of Medicine, University College Cork, Cork, Ireland; School of Pharmacy and Biomolecular Sciences, RCSI University of Medicine and Health Sciences, Dublin, Ireland; School of Pharmacy and Biomolecular Sciences, RCSI University of Medicine and Health Sciences, Dublin, Ireland; Department of Public Health and Epidemiology, School of Population Health, RCSI University of Medicine and Health Sciences, Dublin, Ireland; Department of General Practice, RCSI University of Medicine and Health Sciences, Dublin, Ireland; School of Pharmacy and Biomolecular Sciences, RCSI University of Medicine and Health Sciences, Dublin, Ireland; Department of Public Health and Epidemiology, School of Population Health, RCSI University of Medicine and Health Sciences, Dublin, Ireland; Department of General Practice, RCSI University of Medicine and Health Sciences, Dublin, Ireland; Department of Medicine, School of Medicine, University College Cork, Cork, Ireland; Department of General Practice, School of Medicine, University College Cork, Cork, Ireland

**Keywords:** fall-risk-increasing drugs, prescribing, STOPPFall, falls, older people

## Abstract

**Background:**

Fall-risk-increasing drugs (FRIDs) may contribute to a higher falls-risk in older people, but research examining standardised screening tools is limited.

**Objectives:**

To examine FRID prevalence in community-dwelling older adults, any association with subsequent fall-related hospital admissions, and FRID prescriptions before and after admission.

**Design:**

Retrospective cohort study.

**Subjects/setting:**

Adults aged ≥65 years across 44 general practices in Ireland.

**Methods:**

Fourteen FRIDs, defined by Screening Tool of Older Persons Prescriptions in older adults with high Fall risk (STOPPFall) criteria, were examined as time-varying exposures from 2012 to 2016. The association between FRID exposure and fall-related admissions was estimated using Cox proportional hazards models, adjusted for relevant confounders. Participants’ FRID use pre- and post-fall-related admission was analysed descriptively.

**Findings:**

Of the 35 942 participants, 16 780 (47%) were prescribed at least one long-term FRID at baseline, and 867 (2.4%) experienced a subsequent fall-related admission. Long-term FRID use was associated with a higher risk of fall-related admission [adjusted hazard ratio (aHR) 2.85, 95% CI 2.13–3.82, *P* < .001], demonstrating a stronger association with an increasing FRID burden—one FRID: aHR 2.81 (95% CI 2.07–3.82, *P* < .001); ≥5 FRIDs: 5.67 (95% CI 3.70–8.70, *P* < .001). Long-term FRID prevalence measured pre- and post-fall-related admission was unchanged (53.9%–53.2%). A total of 148 (19.5%) participants had a FRID discontinued, 86 (11.3%) were prescribed an additional FRID and 110 (14.5%) had a FRID initiated for the first time.

**Conclusion:**

FRID use, defined via STOPPFall, was prevalent in older adults and associated with a substantially increased risk of injurious falls. FRID use remained unchanged following discharge. Guidelines recommend including medication review in fall-risk assessments, but further work is needed to embed this across health systems.

## Key points

- Fall-risk-increasing drug (FRID) prevalence, defined by Screening Tool of Older Persons Prescriptions in older adults with high Fall risk (STOPPFall), ranged from 43% to 48% and was associated with an increased risk of subsequent falls.
- Fall-related admission risk increased with the number of FRIDs prescribed and varied across medication classes.
- There was no reduction in the proportion of patients prescribed FRIDs following a fall-related admission.
- This highlights the complexity of deprescribing for older people with multimorbidity and polypharmacy.
- Medication reviews, using STOPPFall, as part of falls risk assessment across primary and secondary care are warranted.

## Introduction

Falls and fall-related injuries are a major cause of morbidity and mortality. They are common in older people with an annual prevalence rate of 30% in Europe [1] and over 26% worldwide [2]. Falls can have a disproportionately severe impact on older adults as they may result in major injury requiring hospitalisation, loss of mobility and independence, early admission to long-term care and a negative impact mental well-being. Due to a globally ageing population, falls and fall-related hospital admissions are predicted to increase substantially in the coming decades [3].

Medication use is a significant modifiable risk factor, with evidence supporting an association between the use of certain medications and falls among older individuals [4]. To mitigate this, clinical guidelines recommend regular structured medication review as an essential component of falls risk assessment and prevention for older adults [5]. Medications that can increase the risk of falls are known as fall-risk-increasing drugs (FRIDs). Many are commonly prescribed in primary care including cardiovascular medications (e.g. antihypertensives) and central nervous system-acting medications (e.g. antidepressants, benzodiazepines) [6]. Although several systematic reviews have examined medication classes that can increase falls risk, until 2020 there was no falls-specific prescribing screening tool for these higher-risk medications to guide clinical decision-making during medication review. Several explicit prescribing indicator sets for potentially inappropriate prescriptions (e.g. Beers Criteria) include medications associated with falls [7]; however, these encompass prescribing issues beyond falls risk and include a large number of medications, making them less suitable for the study objective. Prior research has either examined a limited number of FRIDs, often guided by medication class literature review, or used these broad Potentially Inappropriate Prescribing (PIP) screening tools to identify higher-risk medications [7–9]. In 2020, a fall-specific screening and deprescribing tool was published, called Screening Tool of Older Persons Prescriptions in older adults with high Fall risk (STOPPFall) [6]. This was developed using an international expert Delphi consensus informed by comprehensive literature review and identified 14 medication classes that increase the risk of falls.

In studies to date, STOPPFall medication prevalence is reported as exceeding 40% among community-dwelling adults aged ≥70 years in Europe, with similar rates in Irish cohorts [10–12]. Recent studies have linked STOPPFall medication use to an increased risk of subsequent falls [11, 13–16]. However, existing evidence has methodological limitations (e.g. cross-sectional study design and use of non-time varying exposure methods), use of self-reported falls as outcome measurement and a paucity of primary care-based studies, where most prescribing occurs. A recent systematic review and meta-analysis (*n* = 21 studies) examined changes in prescriptions following fall-related admissions in older people and reported mixed findings, with only one of the included studies using STOPPFall to identify higher-risk medications [17]. The authors highlighted study heterogeneity as limiting the number of studies included in the meta-analysis.

The aims of this study were to examine (i) the prevalence of FRID prescribing in older community-dwelling adults, defined via STOPPFall, (ii) any association with fall-related hospital admissions over time and (iii) any changes in FRID prescriptions pre- and post-fall-related hospital admission.

## Methods

The STrengthening the Reporting of OBservational studies in Epidemiology statement was used in the reporting of this study [18]. This is a retrospective cohort study that included data extracted from 44 participating general practices (GPs) in Ireland between 2012 and 2016. Participating practices were in Dublin (*n* = 30), Galway (*n* = 11) and Cork (*n* = 3). Data were collected using the GP patient Electronic Health Record (EHR) system, Socrates.

### Participants

Community-dwelling adults aged ≥65 years were included. Annual FRID prevalence was estimated among those with ≥1 healthcare interaction (GP prescription, GP consultation or hospitalisation) in each year, while association with first fall-related hospital admission was assessed among participants with ≥1 healthcare interaction during 2012 (baseline year). Eligible individuals were analysed from 1 January 2013, and were followed up until an event occurred, lost to follow-up or the end of the 4-year follow-up period (31 December 2016). To examine any changes in FRID prescribing pre- and post-admission, participants included individuals who had experienced at least one fall-related admission.

### Exposure

The exposure of interest was FRID prescribing, defined via the STOPPFall screening tool. The 14 STOPPFall medication classes are presented in Table 1. For the primary analyses, FRIDs were examined as a composite exposure i.e. 0, 1 and ≥2 FRIDs. Consequently, switching between FRID medication classes was not treated as a change in exposure status. Only prescriptions with a duration ≥28 days were included. Exposure was assessed in each calendar month of follow-up, allowing exposure status to vary over time. Two exposure definitions were applied:

- Long-term FRID use: receipt of FRID prescription(s) across four consecutive calendar months.
- Any FRID use: receipt of a FRID prescription within a single calendar month.

**Table 1:** FRID WHO-ATC codes according to STOPPFall.

FRID	ATC code
Benzodiazepines	N03AE, N05BA and N05CD
Benzodiazepine-related drugs (Z-drugs)	N05CF
Antipsychotics	N05A
Opioids	N02A, R05DA
Antidepressants	N06A, N06CA
Anticholinergics	A03AA07, A03AB05, A03BA01, A03BA03, A03BB01, N04AA01, N04AA02, N04AA04, N04AB02, N04AC01, N02BE5, M03BA03, M03BC01, N05CM05, N05BB01
Antiepileptics	N03
Diuretics	C02L, C03, C07B, C07C, C07D, C08GA, C09BA, C09BX01, C09BX03, C10BX13, C09DA, C09DX01, C09DX03, C09DX06, C09DX07, C09XA52, C09XA54
Alpha-blockers used as antihypertensives	C02CA
Alpha-blockers for prostate hyperplasia	G04CA
Centrally-acting antihypertensives	C02A
Antihistamines	N07CA02, R06
Vasodilators used in cardiac diseases	C01D
Overactive bladder and urge incontinence medications	G04BD, G04CA53

Abbreviations: WHO-ATC = World Health Organisation—Anatomical Therapeutic Chemical.

### Outcome measures

The primary outcome was the first fall-related admission recorded using the International Classification of Diseases 10th Revision (ICD-10) coding system. The following admission codes were included: falls, fractures, head injuries/concussion and syncope/collapse. Hospital discharge summary free-text entries were also examined (see Appendix 1). To examine any changes in FRID prescribing, the outcome of interest was the number of FRID prescriptions following fall-related admission.

### Covariates

Covariates measured at baseline included age, sex, health cover type, number of unique medications, number of fall-related admissions and number of all-cause admissions. The number of FRID-specific medical conditions, as a time-varying covariate, was also included (see Appendix 2). GP-recorded medical diagnoses were coded using the ICD-10 and International Classification of Primary Care Version 2 coding systems. The number of medications prescribed was defined as the total number of unique medications of any duration (including FRIDs) according to the World Health Organisation—Anatomical Therapeutic Chemical (ATC) codes, defined at the ATC 5th level. As health cover type is means-tested, patient eligibility for free/low-cost public medical care was used as an indicator of lower socioeconomic status. Ireland has a mixed public and private system with the General Medical Services (GMS) scheme (means tested based on income) covering ~65% of adults aged ≥65 years in Ireland [19]. Health cover type was classified into two categories based on eligibility for free/low-cost medical care: Public [GMS Scheme, GP (Doctor) Visit Card, National Health Service, European Health Insurance Card] and Private.

### Statistical analysis

Baseline demographic and clinical characteristics were summarised descriptively. Associations between FRID exposure and the first fall-related admission during follow-up were estimated using Cox proportional hazards regression models, with FRIDs as a time-varying exposure. FRID use was examined both as any use and long-term use. Analysis was repeated for fall-related admissions with FRID exposure categorised according to the number of FRIDs prescribed per patient (0, 1, 2, 3, 4, ≥5). All models were adjusted for baseline confounders, including age, sex, health cover type, number of fall-related admissions, number of all-cause admissions, number of unique medications and FRID-specific comorbidity (as a time-varying covariate). To account for clustering of patients within GPs, robust standard errors clustered at practice level were applied. For the primary analysis, zero imputation was used for missing comorbidity data, of which there was 32.5%. Changes in the number of long-term FRID prescriptions before and after a fall-related admission were analysed descriptively at the patient level.

Several sensitivity analyses were conducted. To test the robustness of this analysis, the Cox proportional hazards models examining FRIDs and fall-related admission were repeated with multiple imputation by chained equations for baseline comorbidity data. It was also conducted for each of the 14 STOPPFall-defined FRIDs. Changes in FRID prescriptions before and after fall-related admission for each medication class were also examined. All analyses were conducted using R (version 2024.12.1+563) via the RStudio interface, with missing data reported in the tables.

### Ethical approval

Ethical approval was obtained from the Irish College of GPs Research Ethics Committee on 13 May 2015, for the original study (ICGP_REC_21_0011); a subsequent study amendment for the present study was approved on 6 May 2025.

### Role of the funding source

The funder had no role in the design or conduct of the study, data analysis and interpretation, preparation and review of the manuscript or decision to submit this for publication.

## Results

Table 2 presents the baseline characteristics of the study population (*n* = 35 942) according to long-term FRID use. Annual prevalence rates of long-term FRID use between 2012 and 2016 ranged from 42.8% (*n* = 15 363) to 47.8% (*n* = 17 086), with more than half of these patients prescribed two or more FRIDs (see Appendix 3). The STOPPFall medication classes that were most commonly prescribed long-term were: diuretics (*n* = 9370, 26.1%), antidepressants (*n* = 6859, 19.1%) and benzodiazepine-related drugs (*n* = 4068, 11.3%) (see Appendix 4).

**Table 2:** Study participants’ baseline characteristics according to STOPPFall-defined long-term FRID exposure.

Characteristic^a^	Overall *N* = 35 942	No FRIDs *N* = 19 162	≥1 FRIDs *N* = 16 780
Age in years (mean, SD)	71 (9)	69 (8)	74 (9)
Sex			
Male	15 755 (44%)	9132 (48%)	6623 (39%)
Female	20 148 (56%)	10 003 (52%)	10 145 (61%)
Health cover			
Private	8117 (23%)	5563 (29%)	2554 (15%)
Public	27 722 (77%)	13 534 (71%)	14 188 (85%)
Number of unique medications—2012 (mean, SD)	8 (7)	5 (5)	12 (7)
FRID comorbidity (mean, SD)	1.34 (1.39)	1.02 (1.16)	1.65 (1.51)
Any hospital admission—2012			
0	30 905 (86%)	17 282 (90%)	13 623 (81%)
1	3577 (10.0%)	1427 (7.4%)	2150 (13%)
2+	1460 (4.1%)	453 (2.4%)	1007 (6.0%)
Fall-related admission—2012			
0	35 613 (99%)	19 038 (99%)	16 575 (99%)
1	310 (0.9%)	120 (0.6%)	190 (1.1%)
2+	19 (<0.1%)	4 (<0.1%)	15 (<0.1%)

^a^Number (%) unless otherwise specified missing data: Age = 10 (0.03%), Sex = 39 (0.11%), Health Cover = 103 (0.29%), FRID comorbidity = 11 670 (32.5%).

Table 3 presents the association between STOPPFall-defined FRIDs and first fall-related admission using Cox proportional hazards models. In both unadjusted and adjusted models, FRID exposure was strongly associated with an increased hazard of a subsequent fall-related admission: any FRID exposure, adjusted hazard ratio (aHR) 3.88 (95% CI 2.83–5.31, *P* < .001); long-term FRID exposure, aHR 2.85 (95% CI 2.13–3.82, *P* < .001). Table 3 also stratifies this association by number of STOPPFall-defined FRIDs, with an increase in the magnitude of association reported with an increasing number of FRIDs, albeit with overlapping confidence intervals. There was only a slight attenuation in hazard ratios when multiple imputation by chained equations was used to account for missing comorbidity data (see Appendix 5).

**Table 3:** Cox proportional hazard models for STOPPFall-defined FRIDs and outcome of first fall-related hospital admission.

	Unadjusted any exposure (*n* = 35 942)			Adjusted any exposure^a^ (*n* = 35 793)^c^			Unadjusted long-term exposure^b^ (*n* = 35 942)			Adjusted long-term exposure^a^ ^,^ ^b^ (*n* = 35 793)^c^		
Characteristic	HR	95% CI	*P*-value	HR	95% CI	*P*-value	HR	95% CI	*P*-value	HR	95% CI	*P*-value
FRID use	4.03	3.13, 5.20	**<0.001**	3.88	2.83, 5.31	**<0.001**	3.20	2.53, 4.05	**<0.001**	2.85	2.13, 3.82	**<0.001**
Age				1.06	1.05, 1.07	**<0.001**				1.05	1.04, 1.06	**<0.001**
Female sex				1.38	1.21, 1.58	**<0.001**				1.39	1.21, 1.59	**<0.001**
Public health cover				1.27	0.90, 1.81	0.2				1.51	1.08, 2.10	**0.016**
Number of unique medications				1.03	1.02, 1.04	**<0.001**				1.03	1.02, 1.04	**<0.001**
Comorbidity^d^				0.98	0.91, 1.05	0.5				0.99	0.92, 1.05	0.7
Prior fall-related admission				2.92	2.25, 3.78	**<0.001**				3.08	2.38, 3.98	**<0.001**
Prior all-cause admission				1.08	1.05, 1.11	**<0.001**				1.07	1.04, 1.10	**<0.001**
FRIDs number												
0	–	–	–	–	–	–	–	–	–	–	–	–
1	3.27	2.44, 4.37	**<0.001**	3.52	2.51, 4.93	**<0.001**	2.82	2.18, 3.66	**<0.001**	2.81	2.07, 3.82	**<0.001**
2	3.66	2.81, 4.76	**<0.001**	3.39	2.46, 4.67	**<0.001**	3.11	2.35, 4.11	**<0.001**	2.73	1.97, 3.78	**<0.001**
3	5.33	3.90, 7.29	**<0.001**	4.75	3.28, 6.87	**<0.001**	4.55	3.41, 6.07	**<0.001**	3.80	2.66, 5.41	**<0.001**
4	6.50	4.45, 9.49	**<0.001**	5.55	3.62, 8.52	**<0.001**	4.47	2.97, 6.73	**<0.001**	3.53	2.24, 5.57	**<0.001**
5 or more	7.01	4.62, 10.62	**<0.001**	5.70	3.61, 8.99	**<0.001**	5.68	3.71, 8.71	**<0.001**	5.67	3.70, 8.70	**<0.001**

Abbreviations: CI = Confidence Interval, HR = Hazard Ratio

^a^Zero imputation for was used for missing comorbidity data in adjusted models.

^b^Exposure over four consecutive calendar months (includes a prescription with a duration that covered four calendar months or monthly prescriptions over four consecutive calendar months).

^c^Excludes participants with missing data for age, sex and health cover.

^d^Time-varying covariate.

Bold values indicate statistical significance (p < 0.001).

The prevalence of patients prescribed ≥1 FRID 6 months pre- and post-fall-related hospital admission remained essentially unchanged: 53.9% (*n* = 409) pre-admission and 53.2% (*n* = 404) post-discharge. However, when examining the number of FRIDs per patient from pre- to post-admission, a greater proportion experienced an increase in FRID prescribing (see Table 4). Post-hospital discharge, 148 (19.5%) participants had FRID(s) discontinued, while more had an increase; 86 (11.3%) were prescribed an additional FRID and 110 (14.5%) participants had a FRID initiated for the first time (see Figure 1).

**Table 4:** STOPPFall-defined long-term FRID use pre-admission and post-discharge following first fall-related hospital admission.

Presence of ≥1 FRIDs	Number of participants^a^	**%**
Pre-fall-related hospital admission^b^	409	53.9
Post-fall-related hospital admission^c^	404	53.2
Total number of FRIDs^b^^,^^c^	Number of participants^a^	%
No change		
FRID → FRID (continuation)	175	23.1
No FRID → No FRID	240	31.6
Decreased		
FRID → No FRID (full deprescribing)	115	15.2
FRID → Reduction in FRIDs (partial deprescribing)	33	4.3
Increased		
No FRID → FRID (initiation)	110	14.5
Increase in FRIDs (escalation)	86	11.3

^a^Includes all patients with a fall-related hospital admission and 6 months of data both pre-fall-related hospital admission and post-discharge.

^b^Pre-fall-related hospital admission exposure = long-term exposure (FRID prescriptions for the month of the hospital admission and the three previous calendar months).

^c^Post-discharge FRID burden = uses the 6-month window after discharge month with classification based on: the mode (most frequent category) over the last 3 months only (months 4–6), with a tie-breaker being the last observed month. This approach allowed time for pre-admission prescriptions to be discontinued, if relevant, and provided a measure of longer-term prescribing patterns.

**Figure 1:**
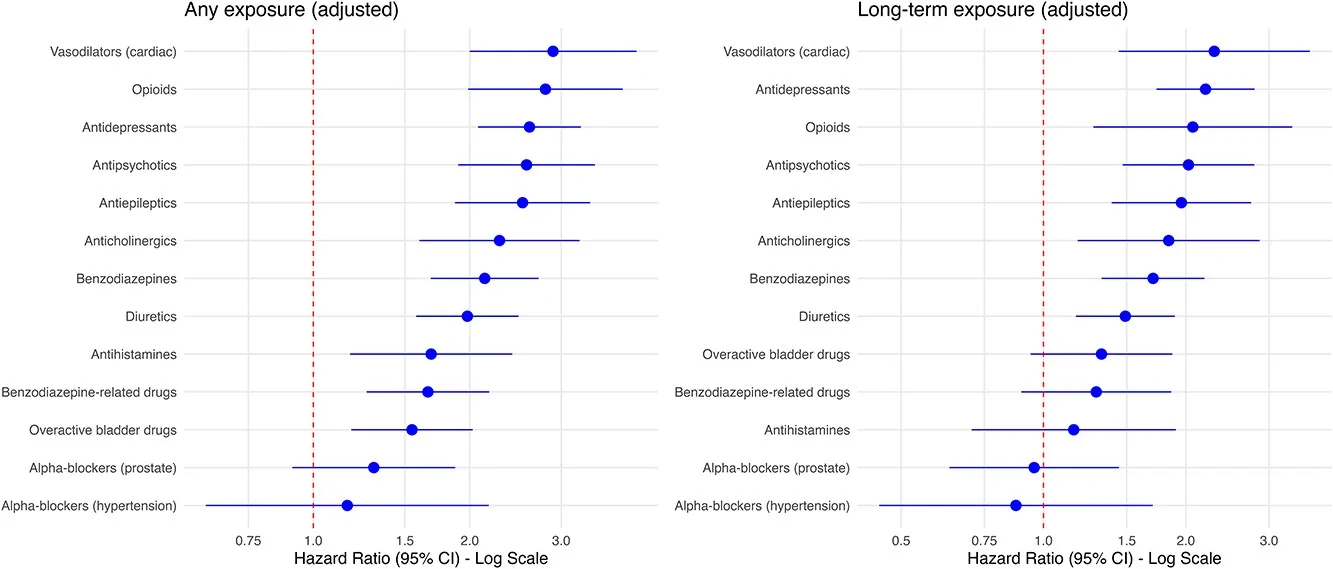
Forest plots for exposure to individual STOPPFall medication classes and first fall-related hospital admission in adjusted Cox proportional hazard models. Note: ^*^Exposure to each individual FRID was compared to no exposure to that specific medication, which could include exposure to other FRIDs. Centrally-acting antihypertensives are omitted from the forest plot as no outcome events ocurred, preventing estimation of a hazard ratio.

Long-term use of eight FRID medication classes was associated with an increase in fall-related admission in adjusted models: benzodiazepines, antipsychotics, opioids, antidepressants, anticholinergics, antiepileptics, diuretics and cardiovascular vasodilators (Appendix 6). The six medication classes that were not associated with fall-related admission included overactive bladder drugs, benzodiazepine-related drugs (Z drugs), antihistamines, alpha blockers (hypertension and prostate) and centrally acting antihypertensives. Some of these medication classes had small sample sizes within this cohort which may affect these estimates (e.g. centrally acting antihypertensives). Three of these (overactive bladder drugs, benzodiazepine-related ‘Z’ drugs and antihistamines) were associated with fall risk when analysed as ‘any FRID use’. Appendix 4 summarises the prevalence of each medication class along with the total number of participants who experienced a fall-related admission. Sensitivity analyses examining changes in FRID prescriptions before and after fall-related admission for each STOPPFall-defined medication class are presented in Appendix 7.

## Discussion

In this large-scale primary care study of older community-dwelling adults, FRID use, as defined by the STOPPFall criteria, was common, with nearly half of all participants prescribed at least one of these medications long-term. Older people prescribed STOPPFall medications were more likely to be admitted to hospital due to a fall. Following fall-related admissions, which may be considered as an opportunity for deprescribing, there was no change in the overall prevalence of FRIDs prescribed for older people when compared to pre-admission.

Our study findings are consistent with previous research that has reported STOPPFall medication use in 42% (*n* = 908) of older community-dwelling adults in a study conducted across five European countries [10]. In the present study, the annual prevalence of STOPPFall medications ranged from 43% to 48% over 5 years. This rate is slightly higher than estimates reported in two previous Irish studies which reported prevalence rates of 42% (*n* = 1198) among older adults using 2009 data [11] and 40% (*n* = 917) in adults aged ≥70 years using 2022 data, both of which used patient interviews to ascertain medication use [12].

Previous international studies examining STOPPFall medications and fall outcomes in older adults have reported lower risk estimates than observed in our study. However, these studies relied on interview-based or self-reported fall outcomes measurement, used different study designs (e.g. cross-sectional, periodic measurement of FRID exposure) and/or were conducted in different care settings (e.g. hospital/pharmacy, population-based surveys) [10, 11, 15, 16]. Of two previous studies that used EHR data to ascertain fall occurrence, one was conducted in an inpatient setting, while the other used outpatient clinic data from one hospital [13, 14]. Our study expands on this by examining this issue in a large-scale primary care patient cohort, including relevant confounders and conducting analyses with a time-varying FRID exposure, increasing the robustness of the findings.

Our study reports that several individual STOPPFall medication classes were associated with fall-related admission, consistent with a systematic review and meta-analysis (*n* = 660 studies) that examined this question [20–22]. Similar to our study findings, opioids, antipsychotics, antiepileptics, antidepressants, benzodiazepines, anticholinergics and (loop) diuretics were associated with fall-related admissions [pooled adjusted odds ratios (aORs) ranging from 1.34 to 2.02], while alpha-adrenoceptor blockers and antihistamines were not. For vasodilator medications, this meta-analysis reported no significant association with falls (pooled aOR 1.14, 95% CI 0.81–1.63), whereas we observed a significant association with long-term use of this medication class and fall-related admission. This meta-analysis included multiple study designs across different study populations with the authors noting substantial heterogeneity and variable study methodological quality. However, as we examined multiple medication classes without correction for multiple comparisons, the magnitude of some associations observed—particularly the finding for vasodilators, where the meta-analysis found no association—may be inflated. This finding should therefore be interpreted with caution and would benefit from examination in other studies. The medication classes that were not associated with fall-related admission may nonetheless increase the risk of falls not resulting in admission. Notably, antihistamines, benzodiazepine-related ‘Z’ drugs and overactive bladder medications were associated with fall risk when analysed as ‘any use’ rather than long-term use, which may suggest acute use of these medications has an impact.

A recent systematic review and meta-analysis (*n* = 21 studies) that examined changes in FRIDs before and after fall-related hospital admission reported mixed findings [17]. Among the nine comparable studies, four reported increases in overall FRID use, four reported no change or mixed findings, while one reported decreased use post-fall. Of the four studies that examined FRID use longer term (3–6 months pre- and post-fall), two reported an increase in use post-hospital discharge [23–26]. The only other study that defined FRIDs using the STOPPFall criteria (*n* = 100) reported a reduction in FRID use from 73% at the time of the fall to 59% 6 months later among older adults attending a fracture clinic in England [27]. Although this suggests greater deprescribing than observed in our study, its prospective design may have influenced prescriber behaviour, introducing observation bias. For our study, it is plausible that the fall itself directly influenced subsequent prescribing, e.g. through initiation of opioids for pain management following a fracture. Additional prescribing may offset any deprescribing of FRIDs which could explain the lack of overall net change observed.

The 2022 World Guidelines for Falls Prevention and Management for Older Adults advocate a multifactorial approach to falls prevention, including the targeting of higher-risk older people for falls-risk assessment [5]. Medication review forms part of this risk assessment and the guidelines recommend use of a validated tool to identify FRIDs. Although PIP screening tools, such as Beers Criteria, already exist, they include a much larger range of medications not directly related to falls [7]. This study demonstrates that STOPPFall medications are associated with injurious falls resulting in hospital admission and supports the case for using STOPPFall in clinical practice to identify higher-risk medications as part of falls risk assessment in older people. Timely transfer of medication data between care settings is essential to enable medication reconciliation, especially following admission to or discharge from hospital. Systematic review evidence indicates that single interventions involving medication review and deprescribing do not appear to reduce the rate of injurious falls [28]. However, existing randomised controlled trials with medication review interventions are heterogenous, thereby limiting the number of trials that can be included in meta-analyses. There is no consensus on the optimal timing or setting for medication reviews for older people.

This study has several strengths. It examined a large-scale primary care cohort of older community-dwelling adults, which increases the generalisability of the findings. It utilised a validated, expert-consensus-derived explicit prescribing screening tool (STOPPFall) to identify FRIDs. Falls leading to hospital admission represent a serious adverse drug reaction, and identification of falls via EHR diagnostic codes rather than self-report reduces recall bias and outcome misclassification. Importantly, STOPPFall medication exposure was defined as a time-varying measure, allowing accurate capture of changes in exposure over time and models were adjusted for relevant confounders, increasing the robustness of the findings. Analyses examined the number of FRIDs at a patient level both pre- and post-admission due to a fall.

This study also had some limitations. Prescribed medications may not fully represent actual medication use, and over-the-counter medicines were not captured. The study period was limited to 2012–16 as this analysis used an existing database. Prescribing practices may have changed since this time; however, the medication classes included are commonly prescribed in primary care. The count of unique medications included all prescribed medicines rather than being restricted to long-term medications. Health cover was used as a proxy measure for socioeconomic status. Comorbidity data were missing for 32.5% of the cohort, which may have introduced bias. However, our primary model additionally adjusted for number of medications (a proxy for comorbidity), all-cause hospital admissions and fall-related hospital admissions; and a sensitivity analysis using multiple imputation yielded essentially unchanged findings. We were unable to assess specific FRID combinations due to insufficient event numbers for reliable estimation; this remains an important area for future research in larger cohorts. We were unable to capture changes in medication dose, meaning deprescribing efforts involving dose tapering rather than discontinuation would not be reflected in the count-based outcome measure of FRID use.

## Conclusion

In this primary care study of older community-dwelling adults in Ireland, FRIDs, as defined by STOPPFall criteria, were prevalent and associated with a substantially increased risk of fall-related hospital admissions over time. Risk differed across individual STOPPFall-defined medication classes and increased with a higher number of fall risk-increasing medications. Despite this, there was no reduction in patients’ FRID use following a fall-related hospital admission. This highlights the complexity of deprescribing for older people with multimorbidity and polypharmacy and the need for health system interventions that prioritise opportunities for regular medication review as part of falls risk assessment and fall-prevention programmes across primary and secondary care.

## Supplementary Material

aa_26_2011_File002_afag303

## Research Data Transparency and Availability

Anonymised data used in this study are available from authors upon request.
