# Supplementary material for: Fall-risk-increasing drug use in older community-dwelling adults: prevalence, association with fall-related admissions and post-discharge prescribing changes—a retrospective cohort study
Source: Age Ageing. 2026 Oct 23;55(10):afag303. doi: 10.1093/ageing/afag303 (PMC13693591; doi:10.1093/ageing/afag303)
Supplement: Supplementary file 1 [file aa_26_2011_file002_afag303.pdf]

Appendices 1-7

Fall-risk–increasing drug use in older community-dwelling adults: prevalence, association with fall-related admissions, and post-discharge prescribing changes — a retrospective cohort study

Table of Contents

Appendix 1: Fall-related hospital admission diagnostic codes and text entries **2**

Appendix 2: STOPPFall FRID-specific comorbidity list **3**

Appendix 3: Findings for annual prevalence of STOPPFall-defined FRID use **5**

Appendix 4: Findings for frequency and prevalence of individual STOPPFall medication classes and first fall-related hospital admissions .. **6**

Appendix 5: Sensitivity analysis of cox proportional hazard models using multiple imputation by chained equations **7**

Appendix 6: Alluvial plot of FRID use pre-admission & post discharge following fall-related admission **8**

Appendix 7: Individual STOPPFall medication class changes pre- and post-fall-related hospital admission **9**

Appendix 1: Fall-related hospital admission diagnostic codes and text entries

Table A1 - Fall-related hospital admission diagnostic codes and text entries

Category	ICD-10 codes	Diagnosis description containing text:
Falls	W00-W19, R29.8, R29.6	"fall" or "fell"
Fractures	S02, S12, S22, S32, S42, S52, S62, S72, S82, S92	"fracture" or "#"
Head injuries / concussion	S06.0-S06.9	“concussion," "head injury," or "traumatic brain injury (TBI)"
Syncope / collapse	R55	"vasovag," "syncope," or "collapse”

Admission diagnoses were manually reviewed for context and excluded if clearly inappropriate (e.g., "lobar collapse," "vertebral collapse," or "pre-syncope" [ICD-10 R42] without an associated fall).[1]

1. Walsh, M. E., Boland, F., Moriarty, F. & Fahey, T. Modification of Potentially Inappropriate Prescribing Following Fall-Related Hospitalizations in Older Adults. *Drugs Aging* 36, 461–470 (2019).

Appendix 2: STOPPFall FRID-specific comorbidity list

Table A2 – STOPPFall FRID-specific comorbidity list (i.e. conditions for which FRIDs are prescribed)

Condition / Diagnosis	ICD Codes	ICPC Codes	Medications (ATC codes)
Anxiety disorders	F40, F41	P74	Benzodiazepines (N03AE, N05BA, N05CD), Benzodiazepine-related (N05CF), Antidepressants (N06A, N06CA)
Depression	F32, F33	P76	Benzodiazepines (N03AE, N05BA, N05CD), Antidepressants (N06A, N06CA)
Sleep disorders (insomnia, other sleep disorders)	G47.0, G47.1	P06	Benzodiazepines (N03AE, N05BA, N05CD), Benzodiazepine-related (N05CF), Antihistamines (N07CA02, R06)
Alcohol and substance use disorders	F10-F19	P15, P19	Benzodiazepines (N03AE, N05BA, N05CD), Antidepressants (N06A, N06CA)
Epilepsy	G40	N88	Antiepileptics (N03), Benzodiazepines (N03AE, N05BA, N05CD)
Schizophrenia and related psychotic disorders	F20, F23, F32.3, F53.1	P72, P98, P99, P76	Antipsychotics (N05A), Benzodiazepines (N03AE, N05BA, N05CD)
Bipolar disorder	F31	P73	Antipsychotics (N05A), Antidepressants (N06A, N06CA)
Dementia	F01, F02, F03	P70	Anticholinergics (A03AA07, A03AB05, A03BA01, A03BA03, A03BB01, N04AA01, N04AA02, N04AA04, N04AB02, N04AC01, N02BE5, M03BA03, M03BC01, N05CM05, N05BB01), Antipsychotics (N05A), Benzodiazepines (N03AE, N05BA, N05CD)
Pain (chronic, neuropathic, other syndromes)	R52, R52.0, R52.1, G89, M79.2, G50	L01-L19, N93, Y77, Y79	Opioids (N02A, R05DA), Antidepressants (N06A, N06CA), Anticholinergics (N02BE5)
Migraine	G43	N89	Antidepressants (N06A, N06CA), Anticholinergics (N02BE5)
Fibromyalgia and related musculoskeletal pain disorders	M79.7, M79.1, M62.4, M62.8	L18, L19, L99	Opioids (N02A, R05DA), Antidepressants (N06A, N06CA), Anticholinergics (N02BE5)
Parkinson's disease and related movement disorders	G20, G21, G24, G25, G25.0	N87, N99	Anticholinergics (N04AA01, N04AA02, N04AA04, N04AB02, N04AC01), Antiepileptics (N03)
Lower urinary tract disorders including neurogenic bladder	N32.8, N32.9, N39.3, N39.4	U02, U04, U20	Overactive bladder (G04BD, G04CA53), Anticholinergics (A03BA01, A03BA03)
Benign prostatic hyperplasia (BPH)	N40	U20	Alpha blockers for prostate (G04CA), Anticholinergics (A03BA01)
Gastrointestinal disorders (IBS, functional GI, other)	K58, K30, A03	D87, D92	Anticholinergics (A03AA07, A03AB05, A03BA01, A03BA03, A03BB01)
Peptic ulcer disease	K25, K26, K27, K28	D84	Anticholinergics (A03AA07, A03AB05, A03BA01, A03BA03, A03BB01)
Vertigo and balance disorders	H81, R42	N17	Antihistamines (N07CA02, R06)

Condition / Diagnosis	ICD Codes	ICPC Codes	Medications (ATC codes)
Spasticity and muscle tone disorders	G83.4, G81	N99, L99	Anticholinergics (M03BA03, M03BC01), Antiepileptics (N03)
Hypertension (primary and secondary)	I10-I15	K86, K87	Diuretics (C02L, C03, C07B, C07C, C07D, C08GA, C09BA, C09BX01, C09BX03, C10BX13, C09DA, C09DX01, C09DX03, C09DX06, C09DX07, C09XA52, C09XA54), Centrally acting antihypertensives (C02A), Alpha blockers (C02CA)
Heart failure	I50	K77, K99	Vasodilators cardiac (C01D), Diuretics (C03, C07B, C07C, etc.)
Coronary artery disease (CAD)	I20, I25	K74, K75, K76	Vasodilators cardiac (C01D), Diuretics (C03, C07B, C07C, etc.)
Valvular heart disease	I34, I35, I36, I37, I38, I39	K83, K99	Diuretics (C02L, C03, C07B, C07C, C07D, C08GA, C09BA, C09BX01, C09BX03, C10BX13, C09DA, C09DX01, C09DX03, C09DX06, C09DX07, C09XA52, C09XA54), Vasodilators cardiac (C01D)
Peripheral artery disease (PAD)	I70, I73	K92	Diuretics (C02L, C03, C07B, C07C, C07D, C08GA, C09BA, C09BX01, C09BX03, C10BX13, C09DA, C09DX01, C09DX03, C09DX06, C09DX07, C09XA52, C09XA54), Vasodilators cardiac (C01D)
Renal disease (acute and chronic kidney disease)	N17, N18	U86, U87, U88, U90	Diuretics (C02L, C03, C07B, C07C, C07D, C08GA, C09BA, C09BX01, C09BX03, C10BX13, C09DA, C09DX01, C09DX03, C09DX06, C09DX07, C09XA52, C09XA54), Vasodilators cardiac (C01D)
Cancer-related pain	C77, C78, C79, C80, Z51.5, R52	Y77, Y78	Opioids (N02A, R05DA), Antidepressants (N06A, N06CA)
Rheumatoid arthritis	M05, M06	L88, L89	Opioids (N02A, R05DA), Anticholinergics (N02BE5)
Autonomic nervous system disorders	G90, I95.1, G90.9	K88, N99	Alpha blockers (C02CA), Anticholinergics (N04AA01, N04AA02, N04AA04, N04AB02, N04AC01)

Appendix 3: Findings for annual prevalence of STOPPFall-defined FRID use

Table A3: Annual prevalence of STOPPFall-defined FRID use in the study period (2012-2016)

		Any Exposure			Long-term Exposure[1]		
**Year**	**N**	**≥1 FRID (%)**	**1 FRID (%)**	**≥2 FRIDs (%)[2]**	**≥1 FRID (%)**	**1 FRID (%)**	**≥2 FRIDs (%)[2]**
2012	35,935	47.7% (n=17131)	23.7% (n=8533)	23.9% (n=8598)	42.8% (n=15363)	22.0% (n=7888)	20.8% (n=7475)
2013	35,727	54.9% (n=19627)	24.8% (n=8859)	30.1% (n=10768)	47.8% (n=17086)	22.2% (n=7928)	25.6% (n=9158)
2014	35,038	53.3% (n=18670)	23.9% (n=8365)	29.4% (n=10305)	46.6% (n=16311)	21.6% (n=7575)	24.9% (n=8736)
2015	34,080	52.2% (n=17783)	23.1% (n=7889)	29.0% (n=9894)	46.0% (n=15664)	21.0% (n=7170)	24.9% (n=8494)
2016	29,170	50.6% (n=14765)	22.1% (n=6452)	28.5% (n=8313)	44.9% (n=13095)	20.5% (n=5980)	24.4% (n=7115)

[1] Exposure over four consecutive calendar months (includes a prescription with a duration that covered four calendar months or monthly prescriptions over four consecutive calendar months)

[2] ≥2 FRIDs over the year; may not be concurrent

Appendix 4: Findings for frequency and prevalence of individual STOPPFall medication classes and first fall-related hospital admissions

Table A4: Summary of frequency and prevalence of individual STOPPFall medication classes and first fall-related hospital admission in Cox proportional hazard models

Medication	Patients (any)	Prevalence of prescriptions (any)	Fall-related admission[1] (any)	Patients (long-term)	Prevalence of prescriptions (long-term)	Fall-related admission[1] (long-term)
Benzodiazepines	5,857	16.3	108	3,892	10.8	75
Benzodiazepine related drugs	5,771	16.1	92	4,068	11.3	63
Antipsychotics	3,511	9.8	62	2,152	6.0	39
Opioids	4,774	13.3	82	2,496	7.0	47
Antidepressants	8,396	23.4	200	6,859	19.1	155
Anticholinergics	4,757	13.2	53	2,093	5.8	30
Antiepileptics	4,494	12.5	98	3,146	8.8	69
Diuretics	10,787	30.0	210	9,370	26.1	151
Alpha-blockers (hypertension)	1,387	3.9	17	1,206	3.4	11
Alpha-blockers (prostate)	2,818	7.9	33	2,351	6.5	21
Centrally acting antihypertensives	92	0.3	0	54	0.2	0
Antihistamines	4,315	12.0	36	1,779	5.0	17
Vasodilators (cardiac)	2,085	5.8	52	1,557	4.3	36
Overactive bladder drugs	2,876	8.0	51	2,214	6.2	36

[1]Number of fall-related admissions that occurred while participant was prescribed specific medication; one event can be across multiple rows if participant had two or more medications

Appendix 5: Sensitivity analysis of cox proportional hazard models using multiple imputation by chained equations

Table A5: Cox proportional hazard models for STOPPFall FRIDs exposure and outcome of first fall-related hospital admission using multiple imputation by chained equations for missing comorbidity data

	Unadjusted Any Exposure (n = 35942)			Adjusted Any Exposure (n = 35793)[2]			Unadjusted Long-term Exposure[1] (n = 35942)			Adjusted Long-term Exposure[1] (n = 35793)[2]		
**Characteristic**	**HR**	**95% CI**	**p-value**	**HR**	**95% CI**	**p-value**	**HR**	**95% CI**	**p-value**	**HR**	**95% CI**	**p-value**
FRID Use	4.03	3.12, 5.20	**<0.001**	3.82	2.81, 5.20	**<0.001**	3.20	2.53, 4.05	**<0.001**	2.82	2.13, 3.75	**<0.001**
Age				1.06	1.05, 1.07	**<0.001**				1.05	1.04, 1.06	**<0.001**
Female Sex				1.39	1.22, 1.59	**<0.001**				1.39	1.22, 1.60	**<0.001**
Public Health Cover				1.27	0.89, 1.80	0.18				1.51	1.08, 2.11	**<0.001**
Number of Unique Medications				1.03	1.01, 1.04	**<0.001**				1.03	1.02, 1.04	**<0.001**
FRID Comorbidity[3]				1.01	0.95, 1.07	0.84				1.01	0.95, 1.08	0.74
Prior Fall Admission				2.86	2.19, 3.74	**<0.001**				3.03	2.33, 3.94	**<0.001**
Prior All-Cause Admission				1.08	1.05, 1.11	**<0.001**				1.07	1.04, 1.10	**<0.001**

[1]Exposure over four consecutive calendar months
[2]Excludes participants with missing data for age, sex and health cover
[3]Baseline covariate
Abbreviations: CI = Confidence Interval, HR = Hazard Ratio

Appendix 6: Alluvial plot of FRID use pre-admission & post discharge following fall-related admission

Figure A6: Alluvial plot of STOPPFall-defined long-term FRID use pre-admission & post discharge following first fall-related hospital admission

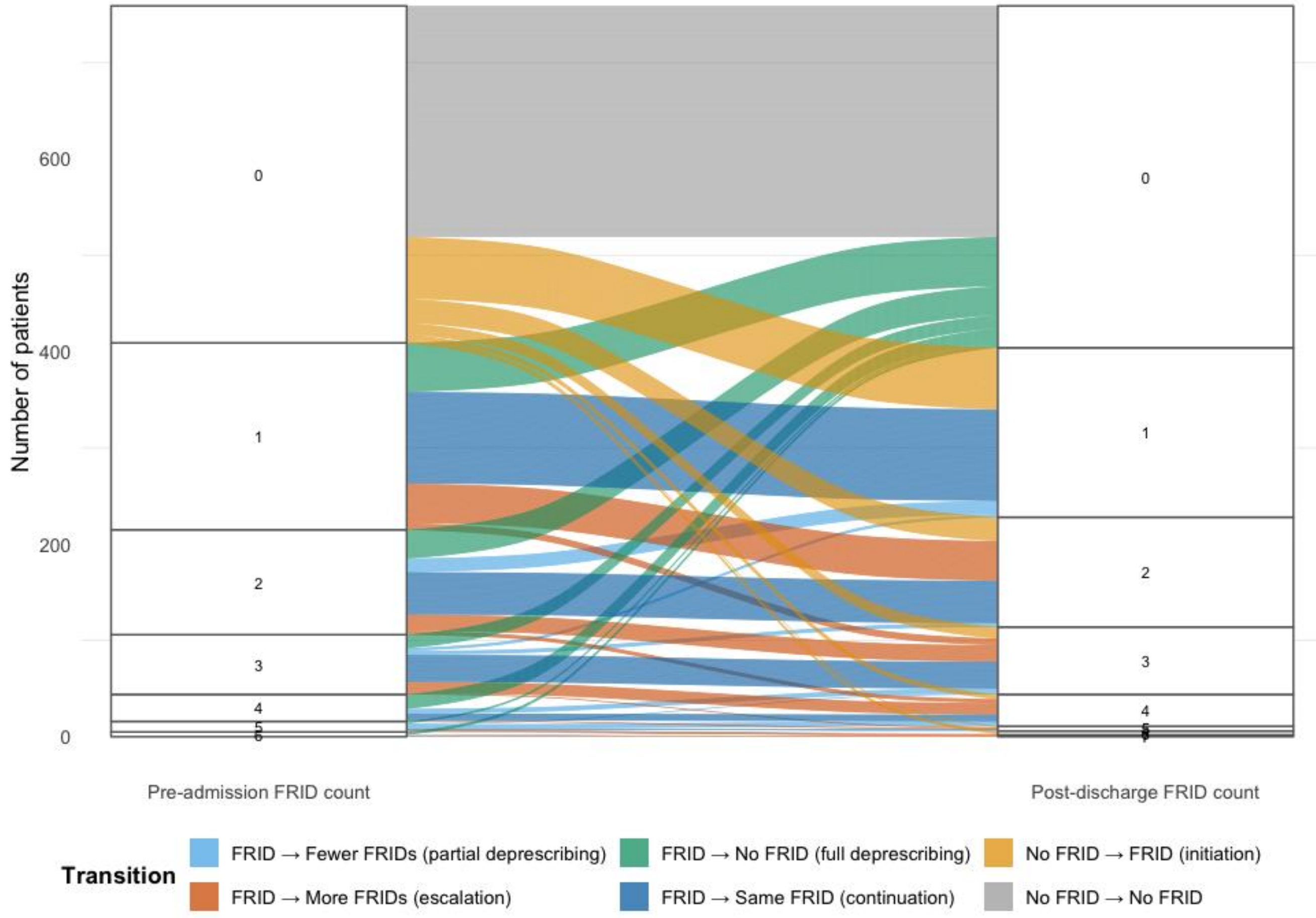

Appendix 7: Individual STOPPFall medication class changes pre- and post-fall-related hospital admission

Table A7 – Individual STOPPFall medication class changes pre- and post-fall-related hospital admission

Pre to Post Admission FRID use	Medication	Medication Count	% of pre-admission prescriptions[1]	% of participants
No Change (continued)	Diuretics	66	40.2	37.7
	Antidepressants	62	38.0	35.4
	Benzodiazepine related	32	47.1	18.3
N=175	Antiepileptics	26	34.2	14.9
	Benzodiazepines	25	32.5	14.3
	Overactive bladder	16	42.1	9.1
	Opioids	15	31.2	8.6
	Antipsychotics	13	31.7	7.4
	Vasodilators (cardiac)	13	36.1	7.4
	Alpha blockers (prostate)	11	47.8	6.3
	Anticholinergics	8	25.8	4.6
	Antihistamines	7	41.2	4.0
	Alpha blockers (hypertension)	6	46.2	3.4
Decreased	Diuretics	74	45.1	50.0
	Antidepressants	65	39.9	43.9
N=148	Benzodiazepines	34	44.7	23.0
	Antiepileptics	34	44.2	23.0
	Benzodiazepine related	29	42.6	19.6
	Opioids	24	50.0	16.2
	Vasodilators (cardiac)	17	47.2	11.5
	Anticholinergics	15	48.4	10.1
	Overactive bladder	15	39.5	10.1
	Antipsychotics	14	34.1	9.5
	Antihistamines	10	58.8	6.8
	Alpha blockers (prostate)	8	34.8	5.4
	Alpha blockers (hypertension)	7	53.8	4.7
Increased	Diuretics	84		42.9
	Antidepressants	80		40.8
N=196	Benzodiazepine related	50		25.5
	Antiepileptics	41		20.9

Pre to Post Admission FRID use	Medication	Medication Count	% of pre-admission prescriptions[1]	% of participants
	Benzodiazepines	39		19.9
	Opioids	38		19.4
	Antipsychotics	27		13.8
	Alpha blockers (prostate)	19		9.7
	Vasodilators (cardiac)	16		8.2
	Overactive bladder	16		8.2
	Anticholinergics	15		7.7
	Antihistamines	12		6.1
	Alpha blockers (hypertension)	4		2.0

[1]Represents the proportion of prescriptions for the medication in the transition group out of all prescriptions of that medication before the fall-related hospital admission (e.g. 40% of diuretics prescribed pre fall-related hospital admission were continued post discharge)
